# High-Performance Bimetallic Electrocatalysts for Hydrogen Evolution Reaction Using N-Doped Graphene-Supported N-Co_6_Mo_6_C

**DOI:** 10.3390/nano14171422

**Published:** 2024-08-30

**Authors:** Renzhe Jin, Shilong Su, Ju Li, Dehai Ping, Yuanyuan Li, Mengyuan He, Xiaomei Yu, Zhengyu Wei, Yong Liu, Songjie Li, Jinyou Zheng

**Affiliations:** 1 School of Chemical Engineering, Zhengzhou University, 100 Science Avenue, Zhengzhou 450001, Chinassl13939572700@163.com (S.S.);; 2 Zhongyuan Critical Metals Laboratory, Zhengzhou University, 100 Science Avenue, Zhengzhou 450001, China; 3 Engineering Research Center of Advanced Functional Material Manufacturing of Ministry of Education, Zhengzhou University, 100 Science Avenue, Zhengzhou 450001, China

**Keywords:** electrocatalysis, hydrogen evolution reaction, bimetallic catalyst, N-doping

## Abstract

Hydrogen has garnered considerable attention as a promising energy source for addressing contemporary environmental degradation and energy scarcity challenges. Electrocatalytic water splitting for hydrogen production has emerged as an environmentally friendly and versatile method, offering high purity. However, the development of cost-effective electrocatalytic catalysts using abundant and inexpensive materials is crucial. In this study, we successfully synthesized nitrogen-doped Co_6_Mo_6_C supported on nitrogen-doped graphene (N-Co_6_Mo_6_C/NC). The catalyst exhibited high performance and durability in alkaline electrolytes (1.0 M KOH) for hydrogen evolution, showcasing an overpotential of 185 mV at a current density of 100 mA cm^−2^ and a Tafel slope of 80 mV dec^−1^. These findings present a novel avenue for the fabrication of efficient bimetallic carbide catalysts.

## 1. Introduction

With the rapid advancement of the socio-economic landscape, the issue of energy scarcity has reached a critical juncture. Hydrogen energy has gained worldwide attention due to its inherent environmental friendliness, high calorific value, and substantial economic potential [1,2,3]. Electrochemical water splitting, as a pivotal technology for hydrogen production, holds significant practical implications [4]. While platinum (Pt)-based catalysts have been regarded as highly efficient catalysts for the hydrogen evolution reaction, their prohibitively high cost and limited availability hinder large-scale commercial production. Consequently, researchers have increasingly focused on exploring economically viable precious metal-free catalysts that are abundant on Earth for use in water splitting applications [5,6,7].

In recent years, the field of water splitting has witnessed significant attention concerning catalysts based on transition metal oxides (TMOs) [8,9,10,11,12,13], including nitrides (TMNs) [14,15,16], sulfides, sulfide catalysts (TMDs) [17,18,19], and phosphides (TMPs) [20,21,22,23]. Among these, transition metal carbides (TMCs) such as Mo, Co, and W have emerged as compelling alternatives to precious metal catalysts for the hydrogen evolution reaction (HER) [24,25]. However, despite continuous efforts, monometallic carbides still exhibit room for improvement in terms of catalytic activity [26]. In recent studies, bimetallic carbides, such as Co_3_Mo_3_C [27,28] and Co_6_Mo_6_C [29,30], have emerged as promising candidates for enhanced activity owing to the inherent metallic bonding between different metal species that facilitates high electrical conductivity.

He et al. conducted the synthesis of Co_6_Mo_6_C supported on graphene oxide [30,31] and reduced graphene oxide [32], which exhibited exceptional catalytic properties in acidic environments. Geng et al. [33] synthesized N-doped bimetallic carbide (N-Co_6_Mo_6_C) nanorods supported on graphene oxide, demonstrating enhanced catalytic activity compared to pristine Co_6_Mo_6_C, which was attributed to electron modulation induced by the incorporation of nitrogen atoms. However, conventional methods for preparing bimetallic carbides typically necessitate annealing temperatures reaching 900 °C or higher. Moreover, in previous studies, additional graphene oxide was required as a carrier to ensure the electrical conductivity of the catalyst material. Unfortunately, the catalysts derived from the aforementioned studies exhibited unsatisfactory performance in alkaline solutions. For N-Co_6_Mo_6_C, the overpotential for achieving 10 mA cm^−2^ was 161 mV in 1 M KOH [33]. However, the Co_6_Mo_6_C catalyst could offer a new insight in the design of a non-noble bimetallic carbide for water splitting.

In this investigation, we present a novel approach for synthesizing N-doped bimetallic carbons possessing porous framework structures, serving as highly efficient electrocatalysts for HER. In contrast to previous methodologies that required additional graphene oxide as a carrier, our study involves the generation of nitrogen-doped graphene (NC) through the calcination of dicyandiamide, while concurrently achieving the formation of N-Co_6_Mo_6_C with enhanced catalytic activity at a lower calcination temperature. The synthesis of N-Co_6_Mo_6_C/NC involves two distinct steps. Firstly, we employ a hydrothermal reaction to prepare CoMoO_4_. Subsequently, dicyandiamide (DCA) is utilized as a carbon and nitrogen source, followed by calcination in an argon atmosphere, resulting in the self-generation of N-graphene-based N-molybdenum cobalt carbide (N-Co_6_Mo_6_C/NC).

## 2. Materials and Methods

### 2.1. Chemicals and Materials

Sodium molybdate dihydrate (Na_2_MoO_4_·2H_2_O,99.9%) was obtained from Kermel. Cobalt nitrate hexahydrate (Co(NO_3_)_2_·6H_2_O, 99.9%), citric acid (CA, AR), dicyandiamide (DCA, 98%), potassium hydroxide (KOH, AR), and ethanol (CH_3_CH_2_OH, AR) were purchased from Sigma-Aldrich, Shanghai, China. Nafion solution (5 wt.%) was purchased from Alfa Aesar, Shanghai, China. This study used all chemicals as received without further purification. Deionized water (>18.25 MΩ cm) was obtained from a Millipore system.

### 2.2. Synthesis of CA-CoMoO_4_

Here, 2 mmol of Co(NO_3_)_2_·6H_2_O and 4 mmol of citric acid (CA) were dissolved in 50 mL of deionized water, followed by stirring for 20 min. Subsequently, 2 mmol of sodium molybdate dihydrate (Na_2_MoO_4_·2H_2_O) was added to the solution, and the mixture was further stirred for 30 min, resulting in an orange-colored solution. The solution was transferred into a 100 mL Teflon-lined stainless-steel autoclave and heated at 200 °C for 10 h. After completion, the reaction mixture was allowed to age for 24 h at room temperature. The resulting purple liquid was filtered, and subsequent washing was performed using deionized water and anhydrous ethanol and repeated three times to remove any unreacted metal ions and impurities. The filtered product was then dried in a vacuum oven at 60 °C for 6 h. The obtained sample was subsequently ground to obtain a blue-violet powder, identified as CA-CoMoO_4_.

### 2.3. Synthesis of N-Co_6_Mo_6_C/NC

A total of 20 mg of CA-CoMoO_4_ and 220 mg of dicyandiamide (DCA) were thoroughly mixed in quartz boat A. Separately, 220 mg of DCA were placed in quartz boat B. Under an argon atmosphere, quartz boat B was positioned upstream, while quartz boat A was placed downstream. The combined system was then subjected to heating, starting from room temperature, with a heating rate of 2 °C min^−1^, reaching a temperature of 500 °C, which was maintained for a duration of 30 min. Subsequently, the furnace temperature was further increased to 800 °C at a heating rate of 5 °C min^−1^ and held constant for 1 h. Additionally, the influence of different calcination temperatures, specifically 700 °C and 900 °C, on the structural characteristics and catalytic activity of the samples was investigated.

### 2.4. Synthesis of CoMoO_4_

The synthesis procedure for CoMoO_4_ followed the same method as that of CA-CoMoO_4_, with the exception that citric acid was not included in the preparation.

### 2.5. Synthesis of CoMo_x_C_y_/C

A mixture comprising 200 mg of glucose and 20 mg of CA-CoMoO_4_ was accurately weighed and thoroughly ground. The resulting mixture was then placed in a quartz boat. The quartz boat, containing the ground sample, was subsequently subjected to a heating process under an argon atmosphere, with a heating rate of 5 °C min^−1^, until reaching a temperature of 800 °C. The reaction was maintained at this temperature for a duration of 3 h. After the completion of the heating process, the sample was carefully removed from the quartz boat and underwent further grinding to obtain a black powder. This black powder corresponds to the modified CoMo_x_C_y_/C material.

### 2.6. Synthesis of N-CoMo_x_C_y_

N-CoMo_x_C_y_ was prepared using the same preparation method as N-Co_6_Mo_6_C/NC, except that CA-CoMoO_4_ was replaced with CoMoO_4_.

### 2.7. Material Characterization

Powder X-ray diffraction (XRD, Karlsruhe, Germany) patterns were obtained on a Bruker D8 Advance diffractometer with a scanning step of 0.02° to examine the bulk crystalline phase of the prepared catalysts. The surface morphology and microstructure of the materials were observed by scanning electron microscopy (SEM, JMS-7500F, Tokyo, Japan), transmission electron microscopy (TEM, Talos™ F200S, Waltham, MA, USA), and high-resolution transmission electron microscopy. The Raman spectra were collected from a Horiba LabRAM HR Evolution (Kyoto, Japan) using a laser (532 nm) as the light source. X-ray photoelectron spectroscopy (XPS, Thermo ESCALAB 250XI, Carlsbad, CA, USA) analysis was performed on a Thermo Scientific Nexsa (ThermoFisher, Waltham, MA, USA) spectrometer.

### 2.8. Electrochemical Measurements

All HER measurements in this work were taken using a Princeton Electrochemical Workstation (AMETEK, Middleboro, MA, USA) with a standard three-electrode device. The Hg/HgO (with 1.0 M KOH) and Pt foil electrodes acted as the reference and the counter electrodes respectively. For the working electrode, 5 mg of catalyst powder was suspended in a solution with 0.99 mL ethanol and 0.01 mL 5 wt.% Nafion, followed by ultrasonication for more than 90 min to form a homogeneous ink. Then, 12 μL of the obtained ink was drop-cast on a glassy carbon electrode (GCE) with a geometric area of 0.0707 cm^2^. We fully dried the surface (loading ~ 0.84 mg cm^−2^). All measurements were performed in an electrolyte solution of 1.0 M KOH, and all potentials were calibrated with a reversible hydrogen electrode (RHE), where E_RHE_ = E_Hg/HgO_ + 0.0592 × pH + *E*^θ^_Hg/HgO_ V. To compare the properties of the prepared catalysts, linear scanning voltammetry (LSV) was performed at a scanning rate of 5 mV s^−1^ with a voltage window from 0.126 to −0.274 V vs. RHE. The polarization curves were corrected with iR compensation regarding the ohmic resistance of the solution. The Tafel slope was measured directly by the electrochemical workstation at a scan rate of 0.1 mV s^−1^ with a voltage window from 0.026 to −0.174 V vs. RHE. The electrochemical surface area (ECSA) data were derived from CV curves at different scan rates. Electrochemical impedance spectroscopy (EIS) was conducted at open circuit potential with a frequency ranging from 10^−2^ to 10^5^ Hz at an amplitude of 10 mV. The stability of the catalysts was studied at a scan rate of 100 mV s^−1^ with a voltage window from 0.126 to −0.274 V vs. RHE for 2000 cycles and chronopotentiometry tests at −10 mA cm^−2^ for 15 h.

## 3. Results and Discussion

**Figure 1:**
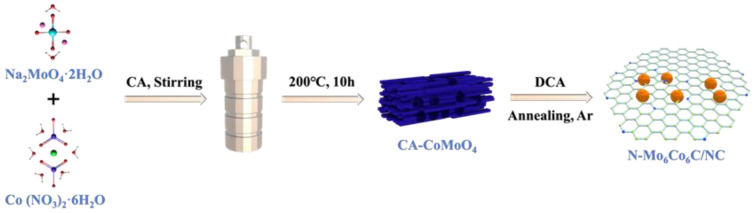
Schematic of the formation of N-Co_6_Mo_6_C/NC.

Figure 1 illustrates the synthesis process of the N-Co_6_Mo_6_C/NC catalyst. Initially, cobalt nitrate hexahydrate (Co(NO_3_)_2_·6H_2_O) and sodium molybdate dihydrate (Na_2_MoO_4_·2H_2_O) were employed as the metal sources for cobalt and molybdenum respectively. In the presence of citric acid, the CA-CoMoO_4_ precursor was synthesized via a hydrothermal method. Subsequently, dicyandiamide (DCA) was used as a carbon source to subject the precursor to high-temperature carbonization at different temperatures (700 °C, 800 °C, and 900 °C), resulting in the formation of N-Co_6_Mo_6_C/NC catalysts. The XRD patterns revealed significant temperature-dependent structural variations in the obtained products (Figure S1a). At 700 °C, diffraction peaks corresponding to CoO (PDF#97-024-5319), Co (PDF#00-015-0806), and Mo_2_C (PDF#00-011-0680) were observed, while the intensities of other peaks were weak, indicating relatively low crystallinity of the material. At 800 °C, the characteristic diffraction peaks of the catalyst matched with the active phase Co_6_Mo_6_C (PDF#03-065-8115), with weak peaks at 2θ values of 39.36° and 48.78° associated with carbon (PDF#97-008-8818). At 900 °C, the XRD spectra can be attributed to Mo_2_C (PDF#00-011-0680), Co_3_Mo_3_C (PDF#97-061-7423), and Co (PDF#00-015-0806), at which time the XRD diffraction peaks become sharp, and the diffraction peaks of Mo_2_C dominate the peaks, and at the same time, due to the high-temperature condition of the material’s sintering and agglomeration, this leads to a decrease in the hydrogen precipitation properties of the material [34,35]. Preliminary electrochemical testing indicated that the N-Co_6_Mo_6_C/NC catalyst prepared at 800 °C exhibited a superior hydrogen evolution capability (Figure S1b). Therefore, a carbonization temperature of 800 °C was chosen for subsequent investigations.

**Figure 2:**
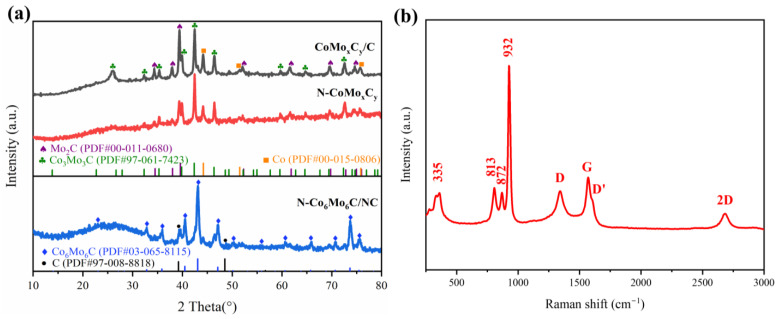
(**a**) XRD patterns of CoMo_x_C_y_/C, N-CoMo_x_C_y,_ and N-Co_6_Mo_6_C/NC. (**b**) Raman spectra of N-Co_6_Mo_6_/NC.

The crystal phase composition was characterized using X-ray diffraction (XRD) patterns (Figure 2a). Figure S2a shows that the precursors CoMoO_4_ and CA-CoMoO_4_ mainly consist of the CoMoO_4_ phase (PDF#97-015-3169) [36,37]. From Figure S2a, it can be observed that when CA was not added, the XRD peaks of CoMoO4 were relatively narrow, indicating good crystallinity and fewer defects. After the addition of CA, the diffraction peaks of CA-CoMoO_4_ became higher and broader, indicating the structural modulation of CA-CoMoO_4_. This suggests an increase in the size and defects of CA-CoMoO_4_, which is beneficial in exposing catalytic sites and effectively enhancing the catalytic activity of the subsequent products. Moreover, the morphology of the precursor transformed from rod-like to defective block-like structures (Figure S2b–e). The characteristic diffraction peaks of the N-Co_6_Mo_6_C/NC catalyst correspond to the active phase Co_6_Mo_6_C (PDF#03-065-8115) and carbon (PDF#97-008-8818). Additionally, the N-CoMo_x_C_y_ and CoMo_x_C_y_/C catalysts exhibit Co, Mo_2_C, and Co_3_Mo_3_C phases. Furthermore, the Raman spectrum of the N-Co_6_Mo_6_C/NC catalyst is consistent with previous reports, indicating the successful synthesis of Co-Mo bimetallic carbides (Figure 2b) [38]. The prominent peaks observed in the Raman spectra of around 932, 872, 813, and 335 cm^−1^ are attributed to CoMoO_4_, indicating the weak oxidation of the bimetallic carbide in ambient air. The band at 932 cm^−1^ corresponds to the symmetric stretching mode of Mo-O bonds. The band at 813 cm^−1^ corresponds to the asymmetric stretching mode of oxygen (O) atoms in O-Mo-O bonds. The band observed at 335 cm^−1^ is attributed to the symmetric stretching of Co-O-Mo bonds. Additionally, the presence of defect features (D peak) at 1341 cm^−1^, the graphitization degree (G peak) at 1571 cm^−1^, and the 2D peak at 2683 cm^−1^ further confirm the existence of graphene components [39]. In addition, the G-peak splits into two peaks of G-peak (1571 cm^−1^) and D-peak (1614 cm^−1^), which is because the localized vibrational modes of the N dopant can interact with the extended phonon modes of graphene, resulting in the observed splitting.

The influence of CA on the material properties was further elucidated through the scanning electron microscopy (SEM) and transmission electron microscopy (TEM) analysis of CA-CoMoO_4_. In Figure S3a–c, rectangular block-like structures can be observed, which are composed of smaller plate-like entities arranged in an ordered manner. The surfaces of these structures exhibit more regular circular pits and cavities with diameters ranging from 1 to 4 μm. Compared to previously prepared smooth surfaces of CoMoO_4_ [11,13,40], these defects are more favorable for the formation of active centers. TEM images in Figure S3e–g provide further insights into the formation process of CoMoO_4_. It can be observed that the presence of numerous curved membranes depends on the strong film-forming ability of CA. Additionally, as a complexing agent, CA can effectively disperse metal salt cations and form long-range metal micelles (CA~MoO_x_-Co^2+^) [14,41]. It is believed that small nanorods are formed during the initial stages of the growth process and that extended hydrothermal conditions may prolong these nanorods.

However, in this experiment, the nanorods were encapsulated by a CA-formed membrane (Figure S3e,f), resulting in the absence of conventional rod-like structures observed in Figure S2d,e. Instead, they underwent continuous aggregation and self-assembly, forming the shape shown in Figure S3e, which consists of nanoscale particles with an approximate diameter of 200 nm. During the subsequent hydrothermal process, these small particles continued to form, accumulate, and self-assemble, resulting in the formation of the block-like material observed in Figure S3a. The folding of the membrane created numerous voids during the accumulation process. The thermal decomposition of CA generated CO_2_, which hindered the crystal growth, leaving behind a significant number of circular pits. As shown in Figure S3g, the membrane is composed of smaller cells (approximately 82 × 300 nm). Scanning electron microscopy images and EDX elemental maps corresponding to Figure S3h further support the formation of CoMoO_4_ and the presence of a carbon film.

**Figure 3:**
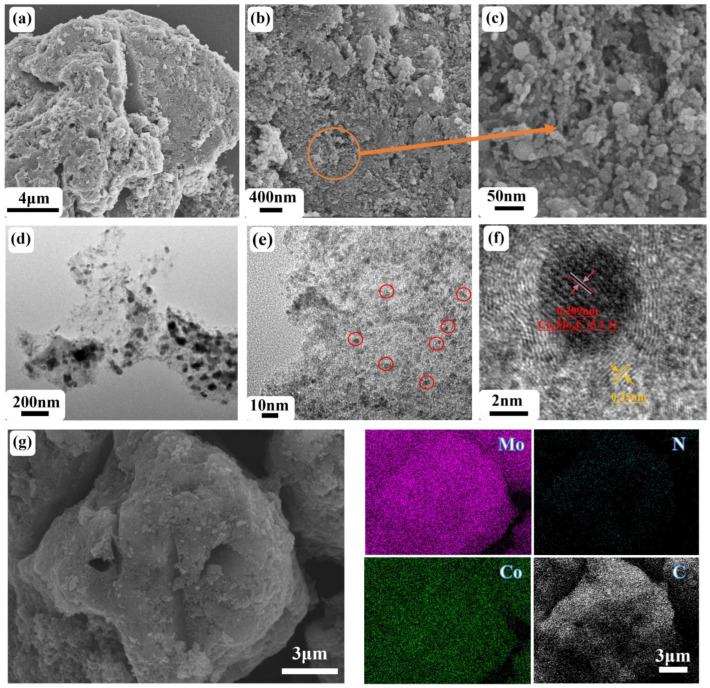
Structure and morphology of the N-Co_6_Mo_6_C/NC. (**a**–**c**) SEM images, (**d**–**f**) HRTEM images, (**g**) SEM image and EDX elemental mapping of Mo, N, Co, and C.

From Figure 3a–c, it can be observed that the catalyst maintains the porous structure of CoMoO_4_ and exhibits a significant presence of dispersed small particles on the surface. These particles range in diameter from 10 to 100 nm, with even smaller particles found on the surface of these small particles. This fractal-like structure allows the catalyst to achieve a larger surface area within a limited volume, thereby exposing more active sites. Due to the carbonization process relying on the thermal decomposition gases of DCA, it is reasonable to expect the growth of similar small particles within the dispersed voids in CoMoO_4_. The HRTEM images in Figure 3d–f demonstrate that N-Co_6_Mo_6_C/NC is formed by the accumulation of active nanoscale particles dispersed on the carbon film. The observed stripe spacing of 0.209 nm in Figure 3f aligns well with the (511) plane of Co_6_Mo_6_C. Furthermore, the slightly larger spacing of 0.35 nm observed in Figure 3f compared to graphene (0.34 nm) may be attributed to the doping effect of N atoms, resulting in an increased interlayer distance [20,42]. The EDX results also confirm the composition of N-Co_6_Mo_6_C/NC, as shown in Figure 3g.

**Figure 4:**
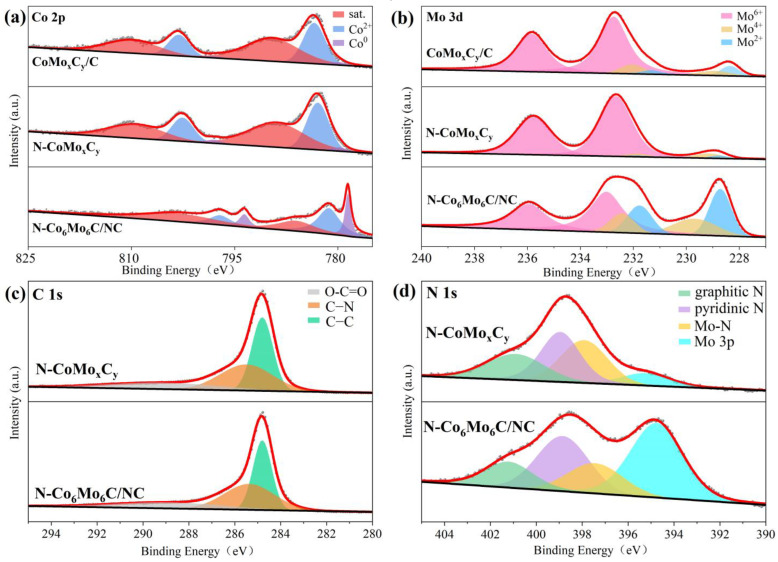
High-resolution XPS spectra of (**a**) Co 2p, (**b**) Mo 3d and (**c**) C 1s for N-CoMo_x_C_y_ and N-Co_6_Mo_6_C/NC. (**d**) N 1s for N-CoMo_x_C_y_ and N-Co_6_Mo_6_C/NC.

X-ray photoelectron spectroscopy (XPS) was employed to chemically characterize the elements present in various catalysts, as shown in Figure S4. The XPS survey spectra revealed the presence of five elements: O, C, N, Co, and Mo. In the Co 2p spectrum of N-Co_6_Mo_6_C/NC (Figure 4a), the peaks at 781.33 eV and 797.17 eV correspond to the Co 2p_3/2_ and Co 2p_1/2_ states of oxidized Co species, respectively, with their satellite peaks located at 786.14 eV and 803.03 eV. The double peaks of Co^0^ at 778.58 and 793.65 eV correspond to the Co 2p_3/2_ and Co 2p_1/2_ orbitals of Co-Co [43,44]. The high-resolution Mo 3d XPS spectrum of N-Co_6_Mo_6_C/NC (Figure 4b) indicates that the peak at 228.72 eV and 231.77 eV corresponds to Mo^2+^, suggesting the presence of a Mo-C bond and the formation of molybdenum carbide [43]. The Mo^2+^ binding energies in N-Co_6_Mo_6_C/NC and N-CoMo_x_C_y_ (228.78 eV, 231.81 eV) are higher than that in CoMo_x_C_y_/C (228.37 eV, 231.34 eV), indicating an increase in Mo-C binding energy upon N incorporation. The peaks at 229.68 eV and 232.43 eV are attributed to Mo^4+^, while the peaks at 233.02 eV and 235.94 eV can be attributed to Mo^6+^. The deconvoluted C 1s spectrum (Figure 4c) reveals peaks corresponding to the C–C bond at 284.8 eV, the C–N bond at 285.4 eV [45], and the O–C=O bond at 288.8 eV. The disappearance of the C=C bond in CoMo_x_C_y_/C and its transformation into the C–N bond in N-Co_6_Mo_6_C/NC and N-CoMo_x_C_y_ confirms the N incorporation [46]. The N 1s spectrum (Figure 4d) shows deconvoluted peaks at 397.35 eV, 398.76 eV, and 401.06 eV, corresponding to Mo-N, pyridinic N, and graphitic N, indicating the uniform doping of N atoms within the carbon matrix [47]. The shoulder peak at 394.72 eV is attributed to the binding energy of Mo 3p. The Mo-N and C-N bonds in the matrix are formed via reactions between Mo and CN_x_ species generated during the subsequent carburization process. Notably, pyridinic N is the dominant N species, playing a crucial role in the HER process, where it can attract electrons and activate hydrogen via single-electron pairs [8,48]. These results strongly confirm the N doping on graphene achieved during the annealing process and the distribution of Co_6_Mo_6_C on graphene.

**Figure 5:**
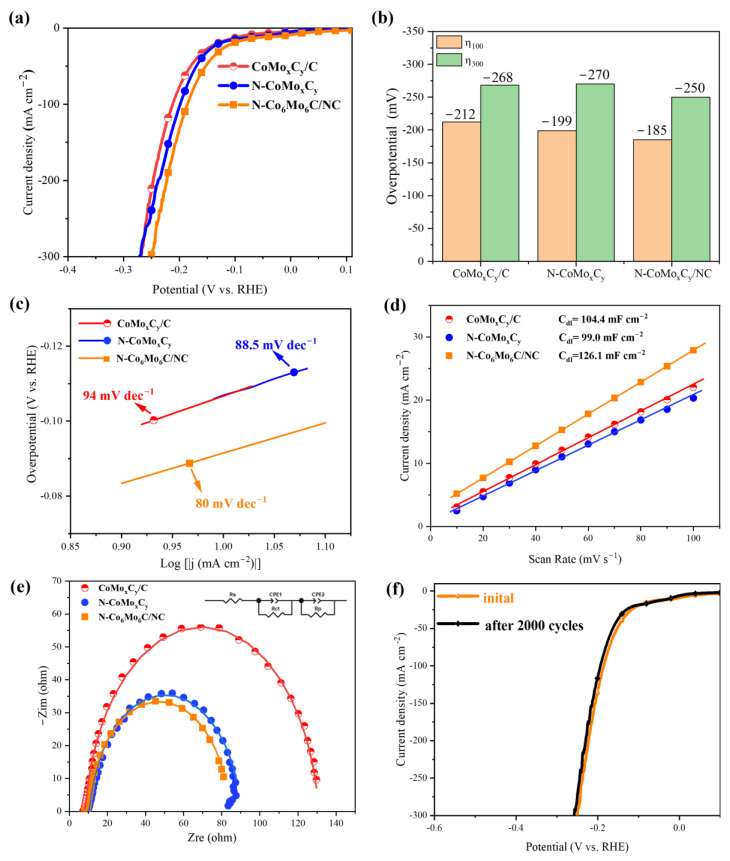
(**a**) iR-corrected LSV curves of HER, (**b**) the corresponding overpotentials (100 mA cm^−2^ and 300 mA cm^−2^), and (**c**) Tafel slopes for CoMo_x_C_y_/C, N-CoMo_x_C_y,_ and N-Co_6_Mo_6_C/NC tested in 1 M KOH. (**d**) Double-layer capacitance (C_dl_) plot of CoMo_x_C_y_/C, N-CoMoxCy, and N-Co_6_Mo_6_C/NC. (**e**) Nyquist plot of CoMo_x_C_y_/C, N-CoMo_x_C_y,_ and N-Co_6_Mo_6_C/NC. (**f**) Durability test of N-Co_6_Mo_6_C/NC.

The electrocatalytic performance of three catalysts for HER was investigated using linear sweep voltammetry (LSV) with iR compensation. As shown in Figure 5a, at a current density of 100 mA cm^−2^, the overpotentials for CoMo_x_C_y_/C, N-CoMo_x_C_y_, and N-Co_6_Mo_6_C/NC were 212, 199, and 185 mV respectively. Generally, the HER in alkaline solutions involves two steps. Firstly, the water dissociates on the catalyst surface, generating adsorbed H atoms and OH, known as the Volmer step (M + H_2_O + e^−^ ⇌ M − H_ads_ + OH^−^). Then, the adsorbed H atoms combine to form H_2_ through the Heyrovsky step (M − H_ads_ + H_2_O + e^−^ ⇌ M + H_2_ + OH^−^) or the Tafel step (2M − H_ads_ ⇌ 2M + H_2_) [18]. From Figure 5c, it can be observed that the Tafel slopes of CoMo_x_C_y_/C (94 mV dec^−1^) and N-CoMo_x_C_y_ (88.5 mV dec^−1^) are similar to each other, but are relatively higher than that of N-Co_6_Mo_6_C/NC (80 mV dec^−1^), indicating the good catalytic activity of N-Co_6_Mo_6_C/NC for HER. The hydrogen evolution process of N-Co_6_Mo_6_C/NC follows the Volmer–Heyrovsky mechanism, where the overall hydrogen evolution reaction is jointly controlled by hydrogen adsorption and electrochemical desorption. The hydrogen evolution performance of this catalyst is superior to most of the reported catalysts (Table S1). Additionally, the charge double-layer capacitance (C_dl_) and the electrochemical surface area (ECSA) were calculated in the non-Faradaic potential region at different scan rates. Figure 5d shows the calculated results, where N-Co_6_Mo_6_C/NC (126.1 mF cm^−2^) exhibits a significantly higher ECSA compared to CoMo_x_C_y_/C (104.4 mF cm^−2^) and to N-CoMo_x_C_y_ (99 mF cm^−2^), indicating that N-Co_6_Mo_6_C/NC possesses a more abundant active center. Electrochemical impedance spectroscopy (EIS) is an effective method for analyzing the kinetics of HER and the charge transfer processes at the electrode/electrolyte interface. From Figure 5e, it can be observed that the three catalysts exhibit similar impedance characteristics, and the inset represents an equivalent circuit based on two-time constant processes. Rs represents the resistance of the solution, the wire, and the contact resistance between the glassy carbon electrode and the catalyst layer. Rs is in parallel with two additional branches: one related to charge transfer processes (CPE1-R_ct_) and the other related to surface porosity (CPE2-R_p_). N-Co_6_Mo_6_C/NC exhibits the lowest R_ct_ (1.3 ohm) and R_p_ (75.62 ohm) compared to CoMo_x_C_y_/C (R_ct_= 6.06 ohm, R_p_= 118.1 ohm) and N-CoMo_x_C_y_ (R_ct_= 1.777 ohm, R_p_= 79.1 ohm), indicating a higher electron transfer rate and a larger surface area for N-Co_6_Mo_6_C/NC, which is consistent with the analysis of the SEM and TEM images of N-Co_6_Mo_6_C/NC. The stability of hydrogen evolution catalysts is an important indicator for evaluating their performance. As shown in Figure 5f, two stability tests were performed on the N-Co_6_Mo_6_C/NC catalyst material. Cyclic voltammetry (CV) tests were conducted, and the polarization curve ƞ_100_ shifted from 185 mV to 194 mV before and after 2000 cycles, indicating the excellent stability of the composite catalyst material. In addition, the Chronopotentiometry experiment was performed at -10 mA cm^−2^ for 15 h in 1 M KOH electrolyte to evaluate stability, as shown in Figure S6. No obvious degradation was observed for N-Co_6_Mo_6_C/NC after 15 h, demonstrating its high stability in alkaline media.

Strategies to improve the electrocatalytic activity of hydrogen precipitation reactions include designing structures to expose more active sites, introducing conductive substrates to accelerate charge transfer [17,23], and doping heteroatoms to modulate the electronic structure of the catalyst [49,50]. This investigation involved the synthesis of CoMoO_4_ with a significant number of defective sites, facilitating the exposure of a greater quantity of active sites. Concurrently, the carbonization process led to the simultaneous generation of numerous active sites within the autogenous graphitic substrate. Notably, these active sites primarily comprise exposed molybdenum atoms in the N-Co_6_Mo_6_C structure. Additionally, the inclusion of pyridine N and graphite N in graphene enhances the electron density of the material, thereby augmenting the electrochemical activity of the hydrogen evolution reaction. Moreover, the crystal structure analysis of N-Co_6_Mo_6_C reveals that each molybdenum atom is connected to six cobalt atoms within the Mo-C octahedral framework. This arrangement effectively reduces the electron density of the unoccupied d orbitals of molybdenum, resulting in a decrease in the excessive hydrogen binding energy of molybdenum. These findings provide a comprehensive explanation for the exceptional electrochemical hydrogen evolution properties exhibited by N-Co_6_Mo_6_C/NC.

## 4. Conclusions

A novel N-Co_6_Mo_6_C/NC with an N-doped graphene substrate was prepared during this work in two simple steps. The experimental results showed that N-Co_6_Mo_6_C/NC, with its excellent conductive substrate and unique structure, contributes to the fast electron transfer between interfaces, the exposure of abundant active sites, and the fast mass and charge transfer. As a result, a large electrochemically active surface area and excellent HER catalytic performance were achieved and exhibited excellent HER activity with an overpotential of 185 mV at a current density of 100 mA cm^−2^. This is the first preparation and study of Co_6_Mo_6_C in electrocatalytic hydrogen precipitation using a simple process, opening up new avenues for the study of nonprecious bimetallic metal-carbon electrocatalysts.

## Data Availability

The data presented in this study are available on request from the corresponding author.

## Supplementary Materials

The following supporting information can be downloaded at https://www.mdpi.com/article/10.3390/nano14171422/s1, Figure S1: XRD pattern and LSV curves of as-obtained catalysts at different pyrolysis temperatures. Figure S2: XRD patterns and SEM images of CA-CoMoO_4_ and CoMoO_4_. Figure S3: SEM images, TEM images, and EDX elemental mapping of the CA-CoMoO_4_. Figure S4: XPS spectra of CoMo_x_C_y_/C, N-CoMo_x_C_y_ and N-Co_6_Mo_6_C/NC. Figure S5: CV curves recorded at different scan rates (10–100 mV s^−1^) within the non-Faradaic potential range for CoMo_x_C_y_/C, N-CoMo_x_C_y_ and N-Co_6_Mo_6_C/NC in 1 M KOH. Figure S6: Long-term stability measurement of N-Co_6_Mo_6_C/NC at −10 mA cm^−2^ for 15 h. Table S1 Performance comparison of N-Mo6Co6C/NC with other HER catalysts in alkaline media. References [51,52,53,54,55,56,57] are cited in the Supplementary Materials.
