# Supplementary material for: High-Performance Bimetallic Electrocatalysts for Hydrogen Evolution Reaction Using N-Doped Graphene-Supported N-Co6Mo6C
Source: Nanomaterials (Basel). 2024 Aug 30;14(17):1422. doi: 10.3390/nano14171422 (PMC11397312; doi:10.3390/nano14171422)
Supplement: Supplementary file 1 [file nanomaterials-14-01422-s001.zip › nanomaterials-3145386-supplementary.pdf]

Supporting Information

High-Performance Bimetallic Electrocatalysts for Hydrogen Evolution Reaction Using N-Doped Graphene-Supported N-Co_6Mo_6C

Renzhe Jin [1], Shilong Su [1], Ju Li [1], Dehai Ping [2], Yuanyuan Li [1], Mengyuan He [1], Xiaomei Yu [1,3], Zhengyu Wei [1], Yong Liu [1], Songjie Li [1,3,*] and Jinyou Zheng [1,3,*]

[1] School of Chemical Engineering, Zhengzhou University, 100 Science Avenue, Zhengzhou 450001, China; ssl13939572700@163.com (S.S.)
[2] Zhongyuan Critical Metals Laboratory, Zhengzhou University, 100 Science Avenue, Zhengzhou 450001, China
[3] Engineering Research Center of Advanced Functional Material Manufacturing of Ministry of Education, Zhengzhou University, 100 Science Avenue, Zhengzhou 450001, China
* Correspondence: songjie@zzu.edu.cn (S.L.); jinyouzh@zzu.edu.cn (J.Z.)

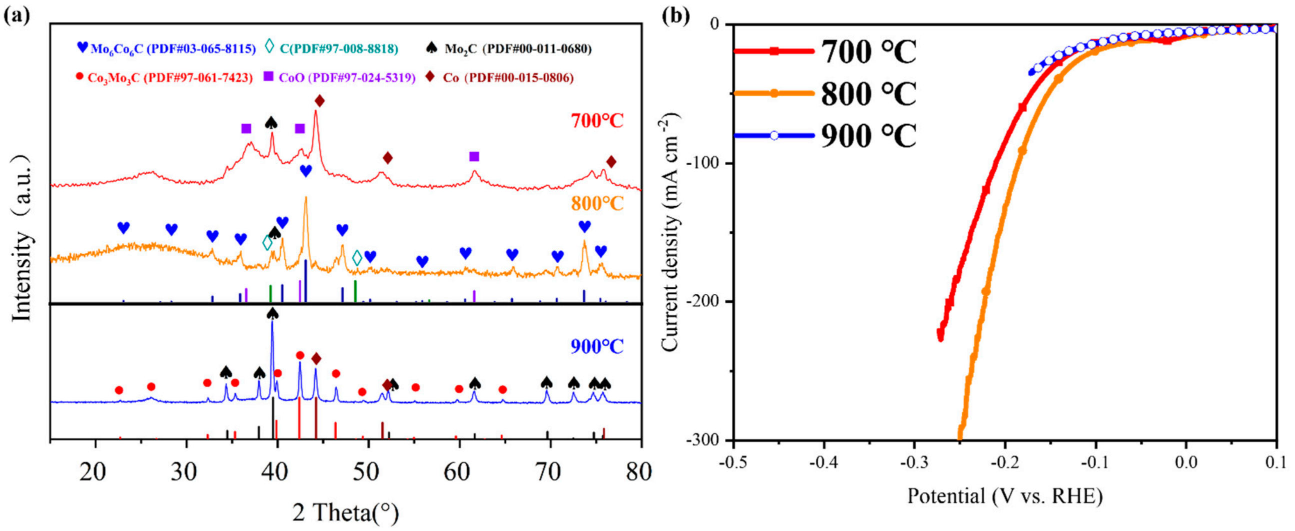

Figure S1 (a) XRD pattern and (b) LSV curves of as-obtained catalysts at different pyrolysis temperatures.

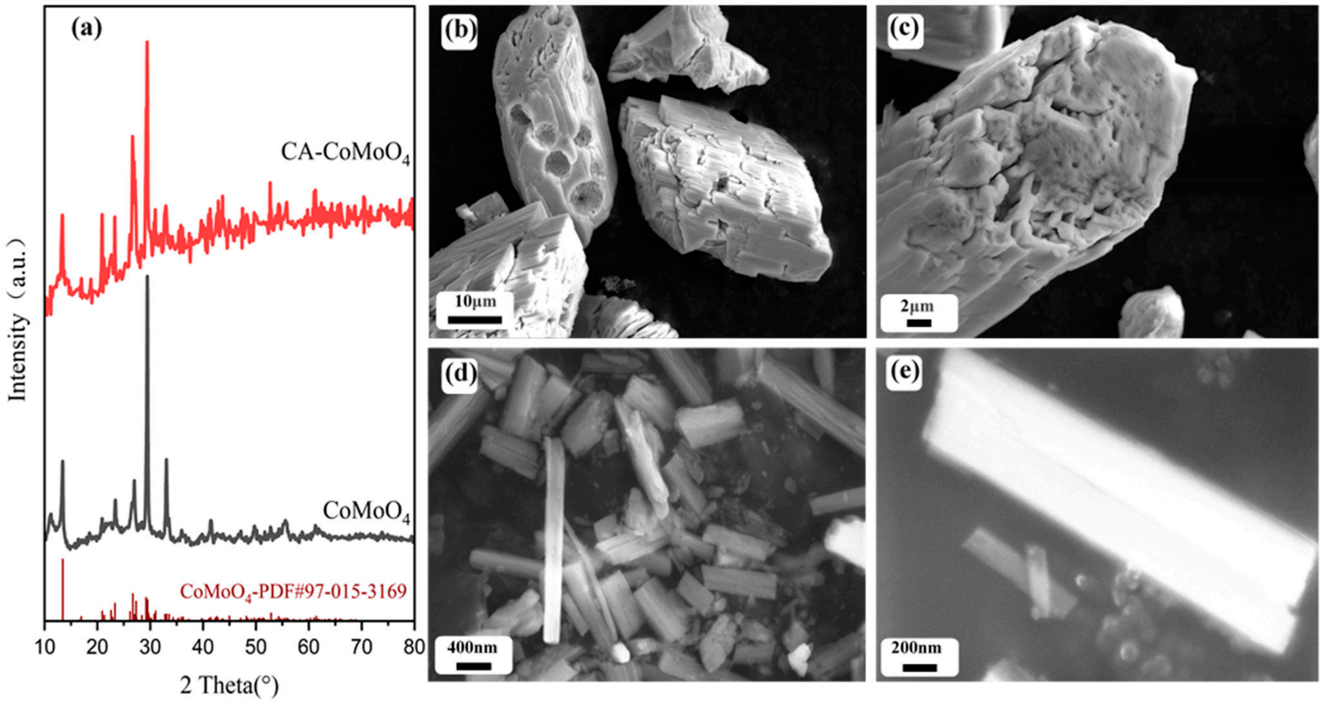

Figure S2 (a) XRD patterns of CA-$CoMoO_4$ and $CoMoO_4$. SEM images of (b-c) CA-$CoMoO_4$ and (d-e) $CoMoO_4$.

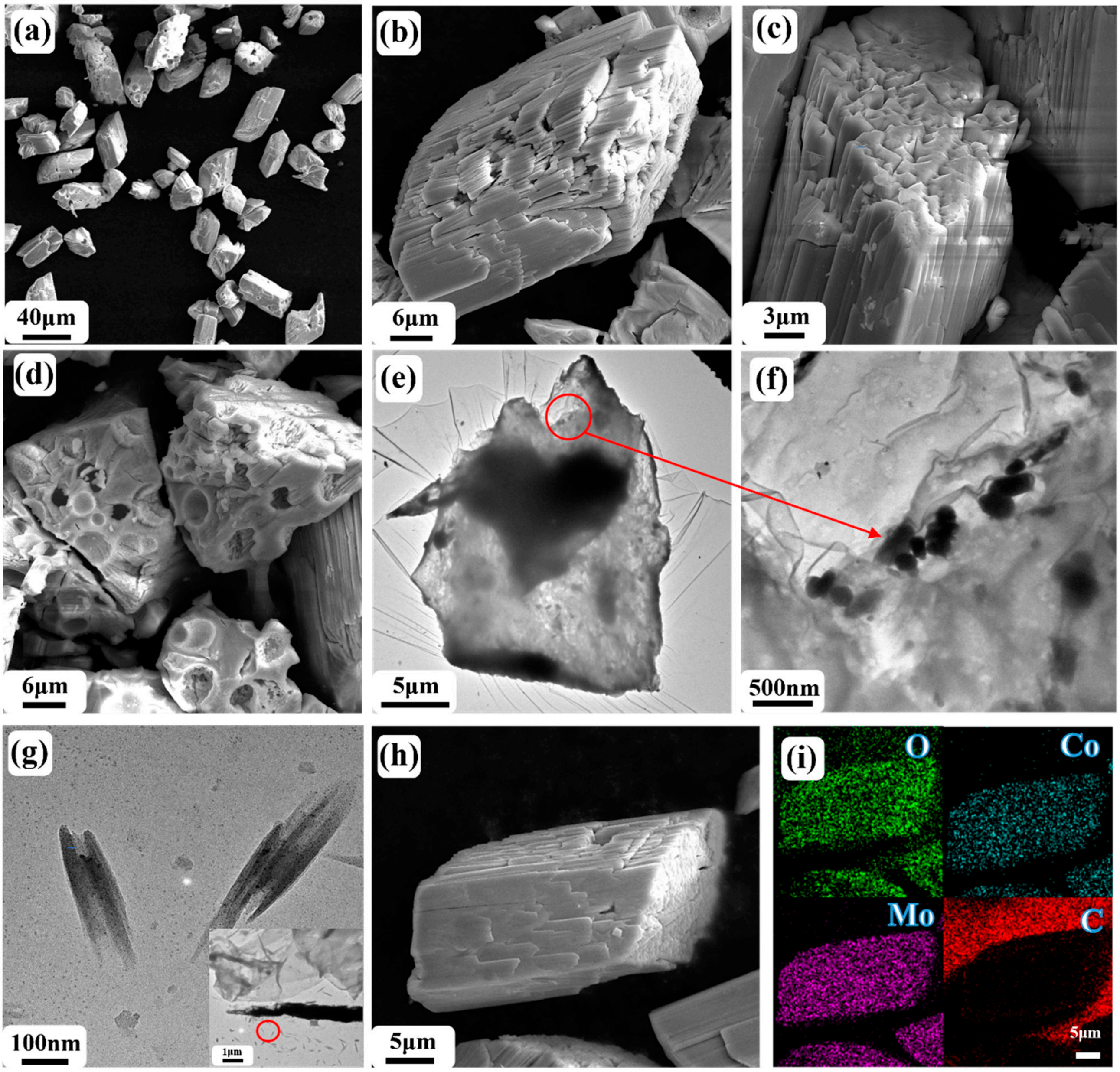

Figure S3 Structure and morphology of the CA-$CoMoO_4$. (a-d) SEM images, (e-g) TEM images, (h) SEM image, and (i) EDX elemental mapping of, O, Co, Mo and C. The inset in (g) depicts the location of (g) with a red box.

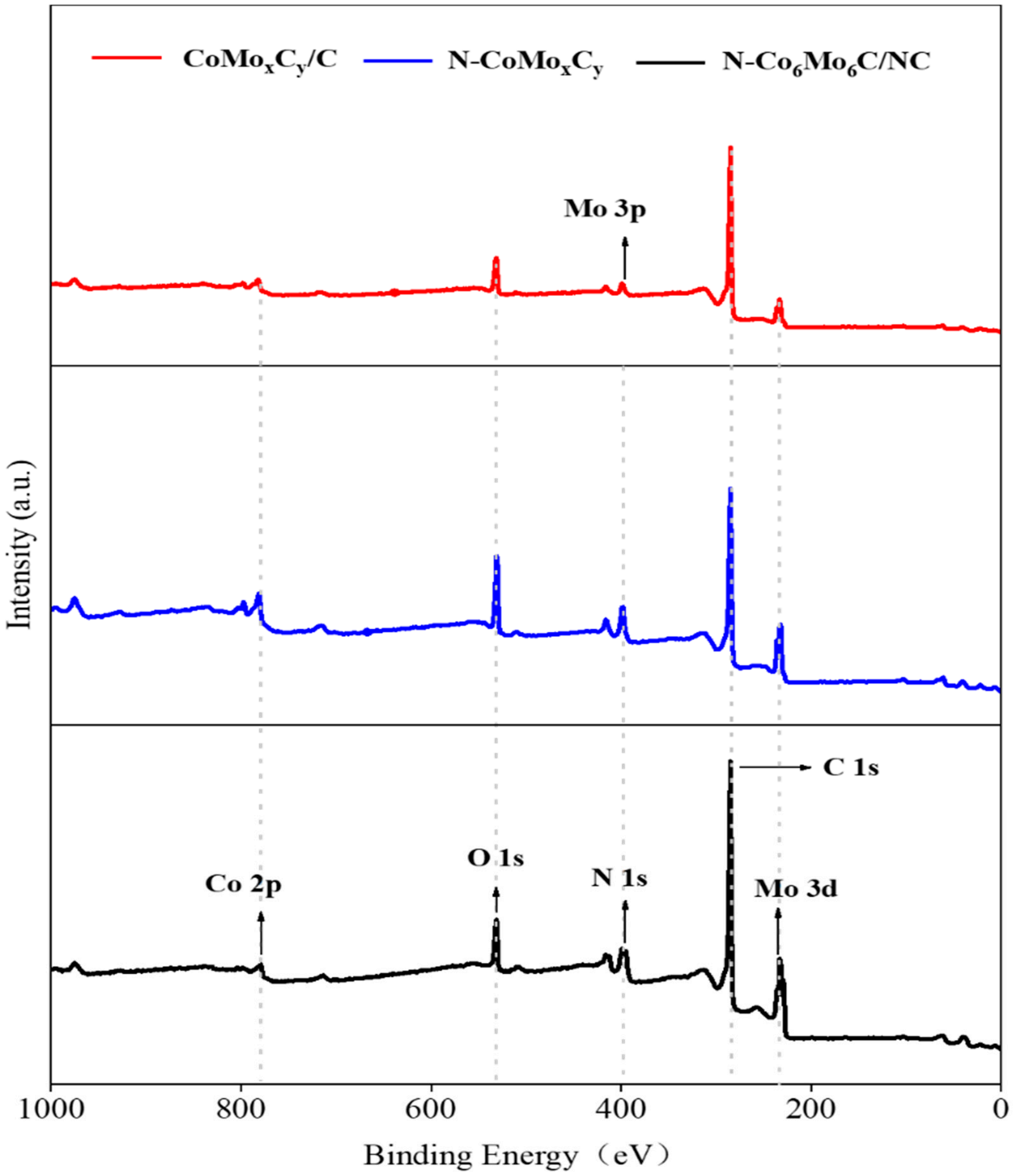

Figure S4 XPS spectra of $CoMo_xC_y/C$, $N-CoMo_xC_y$ and $N-Co_6Mo_6C/NC$.

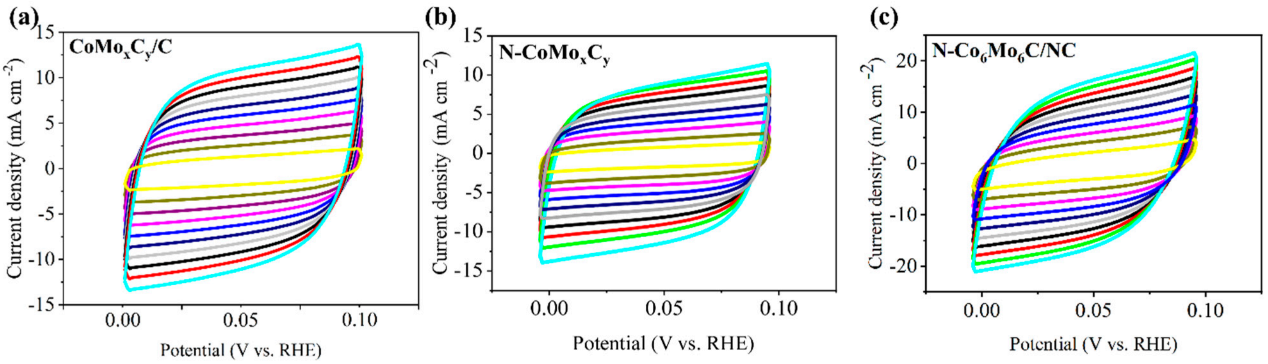

Figure S5 CV curves recorded at different scan rates (10-100 mV s^{-1}) within the non-Faradaic potential range for (a) $CoMo_xC_y/C$, (b) N-$CoMo_xC_y$ and (c) N-Co_6Mo_6C/NC in 1 M KOH.

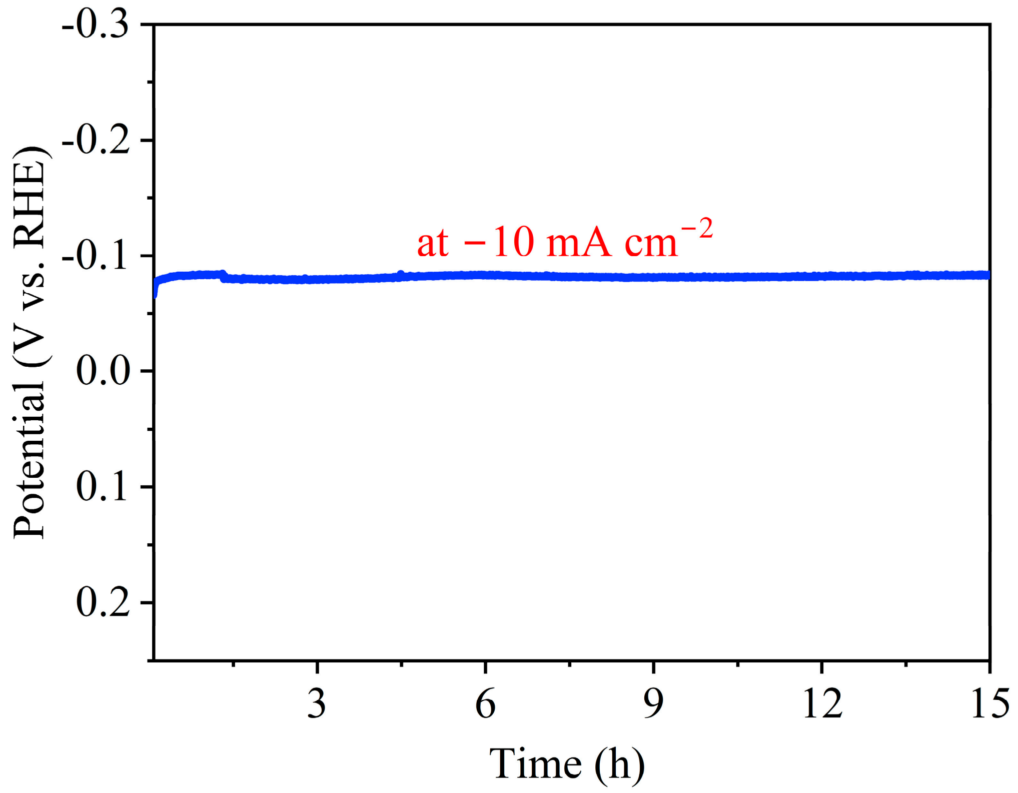

Figure S6 Long-term stability measurement of N-Co_6Mo_6C/NC at −10 mA cm^{-2} for 15 h in 1 M KOH.

Table S1 Performance comparison of N-Mo_6Co_6C/NC with other HER catalysts in alkaline media.

Catalyst	η_{10} (mV)	Tafel slope (mV dec^{-1})	Reference
N-Mo_6Co_6C/NC	10	80	This work
Co/$Co_2Mo_3O_8$/NF	25	33	[1]
CoMoP/C	81	55.5	[2]
CMS-3/NRGO	80	58	[3]
Co-NC/Mo_2C	99	65	[4]
Co/β-Mo_2C/N-CNT	170	92	[5]
CoNC/MoS_2	143	68	[6]
N-doped Co_6Mo_6C	161	76	[7]
Mo_2C/Mo_3Co_3C	87	50.7	[8]
Co_2P/Mo_2C/Mo_3Co_3C@C	154	68	[9]
Mo_3Co_3C/Mo_2C/Co@NC	211	96	[10]

References:

1. Zang, M.; Xu, N.; Cao, G.; Chen, Z.; Cui, J.; Gan, L.; Dai, H.; Yang, X.; Wang, P. Cobalt Molybdenum Oxide Derived High-Performance Electrocatalyst for the Hydrogen Evolution Reaction. *ACS Catal.* **2018**, *8*, 5062–5069, doi:10.1021/acscatal.8b00949.
2. Ma, Y.Y.; Wu, C.X.; Feng, X.J.; Tan, H.Q.; Yan, L.K.; Liu, Y.; Kang, Z.H.; Wang, E.B.; Li, Y.G. Highly Efficient Hydrogen Evolution from Seawater by a Low-Cost and Stable CoMoP@C Electrocatalyst Superior to Pt/C. *Energy Environ. Sci.* **2017**, *10*, 788–798, doi:10.1039/c6ee03768b.
3. Liu, Y.R.; Shang, X.; Gao, W.K.; Dong, B.; Li, X.; Li, X.H.; Zhao, J.C.; Chai, Y.M.; Liu, Y.Q.; Liu, C.G. In Situ Sulfurized CoMoS/$CoMoO_4$ Shell-Core Nanorods Supported on N-Doped Reduced Graphene Oxide (NRGO) as Efficient Electrocatalyst for Hydrogen Evolution Reaction. *J. Mater. Chem. A* **2017**, *5*, 2885–2896, doi:10.1039/c6ta10284k.
4. Liang, Q.; Jin, H.; Wang, Z.; Xiong, Y.; Yuan, S.; Zeng, X.; He, D.; Mu, S. Metal-Organic Frameworks Derived Reverse-Encapsulation Co-NC@Mo_2C Complex for Efficient Overall Water Splitting. *Nano Energy* **2019**, *57*, 746–752, doi:10.1016/j.nanoen.2018.12.060.
5. Ouyang, T.; Ye, Y.-Q.; Wu, C.-Y.; Xiao, K.; Liu, Z.-Q. Heterostructures Composed of N-Doped Carbon Nanotubes Encapsulating Cobalt and β-Mo_2C Nanoparticles as Bifunctional Electrodes for Water Splitting. *Angew. Chemie Int. Ed.* **2019**, *58*, 4923–4928, doi:https://doi.org/10.1002/anie.201814262.
6. Ji, D.; Peng, S.; Fan, L.; Li, L.; Qin, X.; Ramakrishna, S. Thin MoS_2 Nanosheets Grafted MOFs-Derived Porous Co-N-C Flakes Grown on Electrospun Carbon Nanofibers as Self-Supported Bifunctional Catalysts for Overall Water Splitting. *J. Mater. Chem. A* **2017**, *5*, 23898–23908, doi:10.1039/c7ta08166a.
7. Geng, S.; Xu, S.; Yu, Y.S.; Yang, W.; Feng, M.; Li, H. N-Doped Co_6Mo_6C Nanorods as Highly Active and Durable Bifunctional Electrocatalysts for Water Splitting. *J. Electroanal. Chem.* **2020**, *871*, 114271, doi:https://doi.org/10.1016/j.jelechem.2020.114271.
8. Wang, Y.Q.; Xie, Y.; Zhao, L.; Sui, X.L.; Gu, D.M.; Wang, Z.B. Hierarchical Heterostructured Mo_2C/Mo_3Co_3C Bouquet-like Nanowire Arrays: An Efficient Electrocatalyst

for Hydrogen Evolution Reaction. *ACS Sustain. Chem. Eng.* **2019**, *7*, 7294–7303, doi:10.1021/acssuschemeng.9b00358.
9. Li, X.; Wang, X.; Zhou, J.; Han, L.; Sun, C.; Wang, Q.; Su, Z. Ternary Hybrids as Efficient Bifunctional Electrocatalysts Derived from Bimetallic Metal-Organic-Frameworks for Overall Water Splitting. *J. Mater. Chem. A* **2018**, *6*, 5789–5796, doi:10.1039/c7ta10558d.
10. Zhao, Y.H.; Zhang, T.; Wang, X.F.; Li, S.J.; Pan, Y.; Wang, Y.; Song, X.Z.; Tan, Z. Plant Polyphenol-Involved Coordination Assembly-Derived $Mo_3Co_3C/Mo_2C/Co@NC$ with Phase Regulation and Interface Engineering for Efficient Hydrogen Evolution Reaction Electrocatalysis. *New J. Chem.* **2022**, *46*, 13030–13036, doi:10.1039/d2nj01810a.
